# ^1^H-NMR-based metabolomics study of Rifafour in a healthy Kramnik (C3HeB/FeJ) tuberculosis mouse model

**DOI:** 10.3389/fphar.2025.1661938

**Published:** 2025-11-17

**Authors:** Oluwadara Pelumi Omotayo, Siyethemba Bhengu, Yolandy Lemmer, Kobus Venter, Shayne Mason

**Affiliations:** 1 Biomedical and Molecular Metabolism Research (BioMMet), Faculty of Natural and Agricultural Sciences, North-West University, Potchefstroom, South Africa; 2 Future Production and Chemicals, Council for Scientific and Industrial Research (CSIR), Pretoria, South Africa; 3 Preclinical Drug Development Platform at the Faculty of Health Sciences, North-West University, Potchefstroom, South Africa

**Keywords:** tuberculosis (TB), Rifafour, medication, metabolomics, urine, feces, serum, Kramnik (C3HeB/FeJ) mice

## Abstract

Standard treatment for tuberculosis (TB) typically involves the use of four first-line medications, namely, pyrazinamide, isoniazid, rifampicin, and ethambutol. Rifafour is a tablet that consists of a combination of these four anti-TB medications. Immediate TB treatment, typically lasting up to 14 weeks in the hospital as an inpatient and up to 6 months as an outpatient, is imperative to kill the bacteria responsible for TB. However, the anti-TB medication itself is hepatotoxic and can cause several health concerns. To elucidate these metabolic consequences, this study used an untargeted ^1^H-NMR metabolomics approach to investigate the systemic metabolic effects of anti-TB medication administered over 14 days to healthy Kramnik (C3HeB/FeJ) mice – a mouse model commonly used in TB studies. Hippuric acid (p < 0.01), indoxyl sulfate (p < 0.05), phenylacetylglycine (p < 0.01), and tryptophan (p < 0.05) remained significantly decreased in the urine throughout the 14-day TB drug treatment period, whereas in the feces, choline (p < 0.05) and succinic acid (p < 0.01) remained consistently perturbed. Serum collected on day 14 showed significant (p < 0.05) concentrations of glucose, taurine, glycine, uracil, histamine, and allantoin, all of which were upregulated in the TB drug-treated group. This study implies that changes in systemic metabolism directly from TB treatment should be noted and considered when examining animals/patients with active TB on similar TB treatment. That is, these findings highlight the need to distinguish drug-induced metabolic abnormalities from those resulting from infections, consequently aiding the interpretation of metabolomic data in tuberculosis research and improving the development of more accurate therapeutic and diagnostic approaches. Hence, future studies can focus on the perturbed metabolites from TB and account for metabolites resulting from anti-TB medication, as shown in this study.

## 1 Introduction

Tuberculosis (TB) – caused by *Mycobacterium tuberculosis* (*M. tb*) – continues to be a global burden and a public health concern (Farhat et al., 2024). The utilization of multiple TB drugs and the prevalence of multidrug-resistant (MDR) and extensively drug-resistant (XDR) strains of *M. tb* have complicated the management of TB, underscoring the necessity for innovative therapeutic approaches (Deshpande et al., 2024). Moreover, anti-TB medication, especially rifampicin, isoniazid, and pyrazinamide, is extensively shown to induce hepatotoxicity and other deleterious consequences, principally through mechanisms related to oxidative stress, mitochondrial dysfunction, and bile acid alteration (Tostmann et al., 2008; Ramappa and Aithal, 2013). To address this, a fixed-dose combination of TB drugs, such as Rifafour, which comprises rifampicin (150 mg), isoniazid (75 mg), pyrazinamide (400 mg), and ethambutol (275 mg), has been recommended for ease of administration, usage, enhanced bactericidal effect, ability to subdue resistant *M. tb* strains, and reduced treatment duration, among others (Blomberg et al., 2001). Nonetheless, since its introduction, a comprehensive understanding of the metabolic effects and mechanisms of action of Rifafour in the absence of TB infection has not been explored. Employing high-throughput methodologies, such as metabolomics, is crucial for enhancing its application, predicting potential off-target effects (i.e., unintended biological responses or toxicities arising from a drug’s interaction with molecules beyond its primary therapeutic target), and developing effective treatment protocols, given the current deficiency of information in that regard.

Metabolomics, a comprehensive examination of metabolites in biological systems, is a powerful tool for unraveling the biochemical effects of medical interventions (Catussi et al., 2024). Metabolomics is utilized for identifying and interpreting intricate connections within biological systems, aiding in the comprehension of metabolite reactions and relationships (Lin et al., 2024). Through the examination of metabolic changes induced by drug delivery, insights can be gained into therapeutic efficacy, potential side effects of the drug, and the metabolic alterations in biological processes in biofluids (such as serum, plasma, and urine) and tissues (such as the kidney and liver). Proton magnetic resonance (^1^H-NMR) spectroscopy is a prevalent metabolomics technique that is non-destructive, non-invasive, and robust, providing a holistic overview of metabolism (Thirion et al., 2024). This method enables a thorough evaluation of the influence of drugs like Rifafour on the host’s metabolic environment, which is essential for assessing therapeutic benefits and possible adverse effects. Considering the global prevalence of TB and the widespread clinical dependence on Rifafour, it is essential to examine its systemic effects utilizing animal models that accurately replicate human TB pathology.

To date, no study that we are aware of has investigated the metabolic impacts of Rifafour on biofluids, nor is there any research into the interactions and effects of this drug in C3HeB/FeJ (Kramnik) mice. The Kramnik (C3HeB/FeJ) mouse model is hyper-susceptible to pulmonary *M. t*b infection, which is attributed to the presence of an allele, termed the “super susceptibility to tuberculosis 1” (sst1) locus. In addition to other murine models of TB, the Kramnik mouse model of TB exhibits various key features of human TB, including three distinct types of lesions (hypoxic caseous necrotic lesions, disorganized neutrophilic lesions, and cellular lesions) and extracellular and intracellular hypoxic mycobacteria of different phenotypes (Driver et al., 2012). This mouse model is valuable because it enables us to investigate the metabolic changes induced by TB. Moreover, evaluating the effect of Rifafour on this mouse model will help us understand the underlying effects of TB treatment on patients (Li et al., 2025). This study aims to collect data on the modified global metabolic profile caused by Rifafour and to elucidate the metabolic dynamics associated with this drug. That is, to elucidate the metabolic alterations induced by Rifafour in C3HeB/FeJ mice, thereby offering valuable insights into TB therapy. By focusing on the non-infected animal, we aim to clarify the drug’s pharmacological effects on metabolism, which may later inform future studies on its application in TB treatment and assist in the development of personalized therapeutic regimens. Hence, this study employs a ^1^H-NMR metabolomics approach to investigate the changes in metabolic profiles of urine, serum, and feces of C3HeB/FeJ mice administered a 14-day course of Rifafour.

## 2 Materials and methods

### 2.1 Chemicals

Deuterium oxide and trimethylsilyl-2,2,3,3-tetradeuteropropionic acid (TSP) were acquired from Merck (Darmstadt, Germany). Potassium phosphate monobasic (KH_2_PO_4_) and potassium hydroxide (KOH) were acquired from Sigma-Aldrich (St. Louis, Missouri, United States).

### 2.2 Animals

Animal handling and experiments were conducted following the guidelines for animal experimentation of North-West University, South Africa. The study/experiment design and protocol were approved by the North-West University Animal Care, Health, and Safety Research Ethics Committee (NWU-AnimCareREC) with the approval number: NWU-00785-23-A5. A total of 14 C3HeB/FeJ mice (consisting of eight females with an adult body weight range between 18 g and 20 g, and six males with an adult body weight range between 22 g and 23 g) were bred and enrolled for the study at 8 weeks of age. All experimental animals were reared and housed at the North-West University (NWU) vivarium (SAVC reg. no. FR15/13458; SANAS GLP compliance no. G0019; AAALAC accreditation international file #1717) of the NWU Pre-Clinical Drug Development Platform (PCDDP). The animals were housed in well-ventilated cages under standard conditions. Mice were housed four per Techniplast GM500 individually ventilated cage (391 mm width, 199 mm depth, and 160 mm height) under positive pressure of 75 air changes per hour, cage temperature of 20–24 °C, and cage relative humidity of 40%–70%. The animal room received HEPA-filtered air under positive pressure, with 23 air changes per hour. The room temperature was maintained at 22 ± 2 °C, the room relative humidity was 55% ± 15%, and the light/dark cycle was set at 12 h each. Sterile corn cobs were used for bedding, supplemented with paper towels for nesting material and PVC pipes for environmental enrichment. Mice had *ad libitum* access to water and food (standard chow) and were weighed and evaluated before any intervention for baseline clinical signs.

The mice were allowed to acclimatize for 5 days before the start of the experiment or before any intervention. All procedures performed on the animals were following the ethics code for research, training, and testing in South Africa and complied with national legislation. The animals were monitored throughout drug administration (Rifafour) and before sample collection. Particular attention was given to alleviating possible distress and pain by implementing environmental enrichment, rigorous health monitoring, and humane handling techniques, therefore safeguarding animal welfare during the study (no definitive pain or distress was observed).

The mice were assigned to two treatment groups using a block randomization technique, with each group consisting of seven mice. Within each cage, animals were randomly assigned to receive either Rifafour or a water control to maintain balanced group sizes throughout the enrollment period. Block randomization and blinded analysis were done. For 14 consecutive days, each mouse received an oral dose of Rifafour via oral gavage, while the control group received water only. That is:

Group 1 – Rifafour.

Group 2 – no intervention (water only).

Rifafour tablets were prepared daily for delivery by suspending them in distilled water (vehicle). A Rifafour tablet (900 mg) was dissolved in 30 mL of distilled water (30 mg/mL) and sonicated to ensure complete dissolution. The suspension was vortexed extensively to achieve uniformity and was delivered promptly after preparation to avert degradation of active chemicals. The gavage dose volume was calibrated based on the body weight of each mouse–at 10 µL per gram body weight, this equated to approximately 200 µL per mouse (∼20 g).

After drug administration, all experimental animals were placed in metabolic cages for an average duration of 6 h for the collection of urine and fecal samples on days 1, 3, 7, and on the 14th day, the last day of the experiment. To ensure that the drug had reached its peak concentration and saturated the tissues of the mice, urine and fecal samples were collected up to 6 h after administering Rifafour on each of the four collection days (days 1, 3, 7, and 14). On the 14th day, the animals were exsanguinated by transection of the carotid arteries and jugular veins, after which blood samples were collected. Serum samples were immediately obtained from the whole blood by centrifugation (15,000 x *g* for 5 min). All individual mouse samples were stored at −80 °C until metabolomics analysis.

### 2.3 Sample preparation

According to the described methods of Van Zyl et al. (2020), 20.4 g potassium phosphate monobasic (KH_2_PO_4_) was dissolved in 80 mL deuterium oxide to a concentration of 1.5 mM to make an NMR buffer solution (pH 7.4), followed by the addition of 100 mg trimethylsilyl-2,2,3,3-tetradeuteropropionic acid (TSP; internal standard). Fifty-four (54 µL) of urine samples and 6 µL of NMR buffer were used, following the programmed pipetting sequence of the eVol^Ⓡ^ NMR digital syringe as described by Van Zyl et al. (2020). 54 μL of the urine sample was then transferred to a 2 mm NMR glass tube.

Following the lead of Tong et al. (2022), approximately 100 mg of fecal samples was added to 1,000 µL of distilled water (ddH_2_O) and vortexed for 1 min. The mixture was sonicated in a water bath for 30 min and centrifuged at 13,000 x *g* at 4 °C for 15 min 540 μL of the supernatant was transferred into a microcentrifuge tube, and 60 µL of buffer containing TSP was added. The mixture was vortexed for 20 s, centrifuged at 13,000 x *g* at 4 °C for 15 min to remove macromolecules, and 540 μL of the supernatant was transferred to a 5 mm glass NMR tube and sealed with a cap.

Serum samples were prepared following the method of Mason et al. (2018) – 1 mL of serum sample was added into pre-rinsed centrifugal units and centrifuged at 3,000 x *g* for 30 min 300 μL of the filtrate was collected and added to 300 μL of buffer solution in a microcentrifuge tube. The mixture was vortexed and centrifuged at 12,000 x *g* for 5 min, and 540 μL supernatant was collected and transferred into a 5 mm NMR tube and sealed with a cap.

### 2.4 ^1^H-NMR analysis

All samples were randomly loaded onto a SampleXpress autosampler while quality control samples (QCs; pooled volumes of each sample type) were placed at intervals. The analysis was conducted on a Bruker Avance III HD NMR Spectrometer at 500 MHz. The settings for the NMR analysis were similar to those of Van Zyl et al. (2020) for the 2 mm NMR tubes analysis, as well as that of Davoren and Mason (2023) for the 5 mm NMR tube analysis. The temperature of the samples was maintained at 300 K, and the H_2_O resonance peak was suppressed through single-frequency irradiation (NOESY) during a relaxation delay of 4 s, initiated at 90° excitation pulse for 8 µs. Shimming was done automatically on the deuterium signal. Fourier transformations, phase, and baseline correction were also conducted automatically. Verification of the spectral quality was done by ensuring that the resonance line width for TSP was less than 1 Hz. For data processing, Bruker Topspin (v3.6) and Bruker AMIX (v3.9.14) were used for data processing, binning, metabolite identification, and quantification, respectively. Selective binning was performed on the serum data by selecting regions with discernible peaks and excluding noise regions and exogenous compounds to create a data matrix of 135 bins. The urine and fecal samples underwent set-width binning (0.02 ppm intervals), resulting in 456 and 462 bins for the urine and feces, respectively. All spectral data sets excluded the region of the water peak at approximately 4.70 ppm.

### 2.5 Statistical analysis

The ^1^H-NMR spectra for serum and feces were manually baseline and phase corrected and referenced to the TSP signal, while the urine spectra were referenced to creatinine to account for urine dilution differences. MetaboAnalyst 6.0 (www.MetaboAnalyst.ca/), an online suite for metabolomics statistical analysis, was used to examine the spectral data sets for multivariate statistical analysis. To identify outliers and general trends, as well as to examine group clustering, principal component analysis (PCA) was used to visualize the data. Supervised partial least squares discriminant analysis (PLS-DA) was also performed to identify metabolic variations associated with the drug. The R^2^ parameter was used to assess the goodness of fit, while the Q^2^ parameter was used to evaluate the predictive ability of the PLS-DA model. To discriminate the variables that contributed to sample clustering among groups, the variables of importance in the projection (VIP) values of all the peaks from the PLS-DA models were analyzed to identify important metabolites. All multivariate analyses were performed on parametric data, which were log-transformed and Pareto scaled. A library of pure compounds was used to identify significant metabolites. For univariate comparisons of individual metabolites, we did not presume normality and hence employed nonparametric tests. Nonparametric data were used to calculate the p-values (t-test) for the identified VIP metabolites. In this study, *a priori* cutoff VIP value >1 and FDR p-value <0.05 were used to identify statistically significant metabolites. Hedge’s effect size was calculated per time comparison, with a large effect size being a Hedge’s g-value >0.8.

## 3 Results

### 3.1 ^1^H-NMR results of serum

**FIGURE 1:**
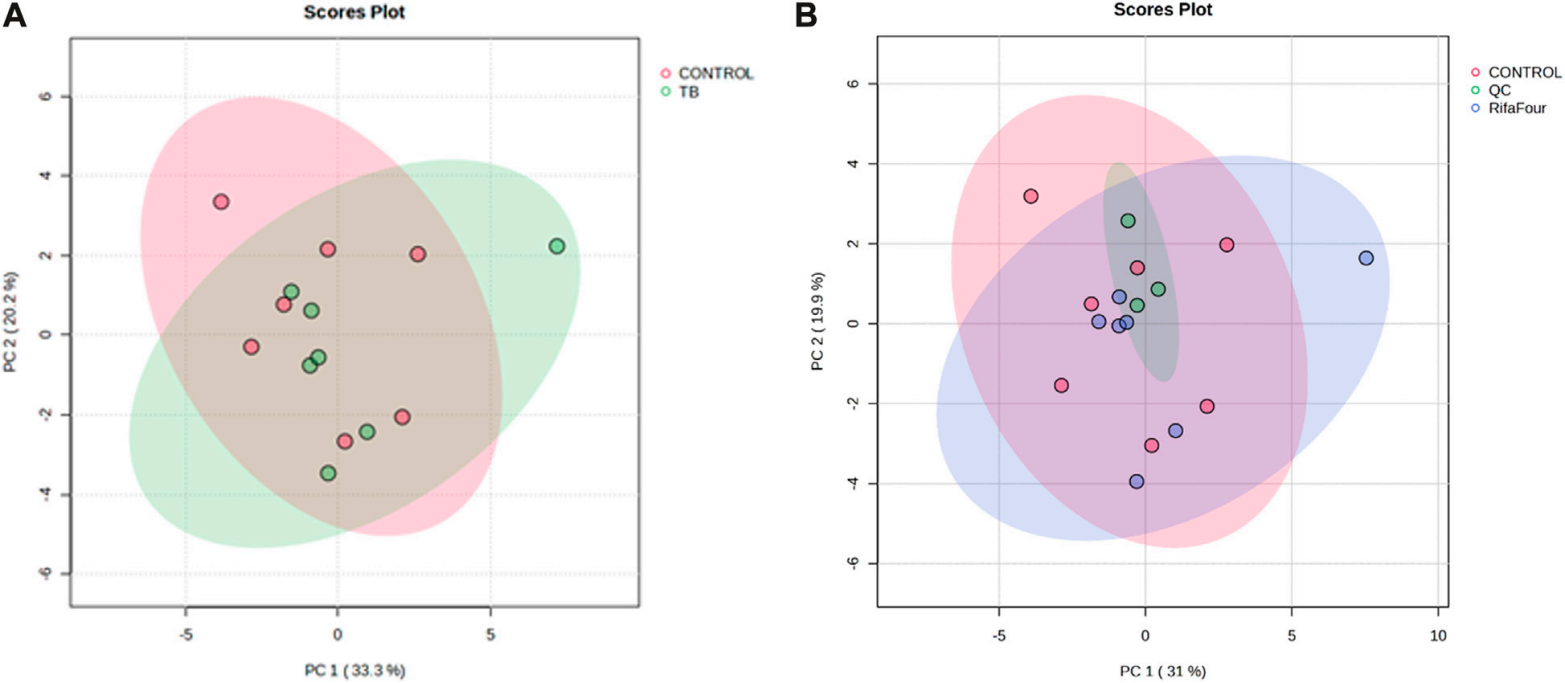
PCA score plots of serum samples collected on day 14. **(A)** Control group vs. Rifafour group, with considerable overlap between the two groups. **(B)** Inclusion of quality control samples (QCs) for quality assurance (QCs cluster together, indicating reliable analytical data).

Figure 1A shows the PCA scores plot of serum samples (control and TB treatment) on day 14 after administration of Rifafour. No outliers (no samples lying outside the 95% confidence ellipses) are evident. Figure 1B includes the QCs, which cluster closely together, indicating negligible analytical drift in both the analysis and data acquisition (i.e., reliable analytical data). The serum PLS-DA model demonstrated a modest fit (R^2^ = 0.40) with marginal predictive capability (Q^2^ = 0.50). Although these values indicate some discrimination across treatment groups, the model exhibited insufficient predictive reliability; hence, interpretation relied predominantly on univariate data (FDR p < 0.05), which identified the following significant metabolites: glucose, taurine, glycine, uracil, histamine, and allantoin, all of which were upregulated in the TB drug-treated group.

### 3.2 ^1^H-NMR results of urine

**FIGURE 2:**
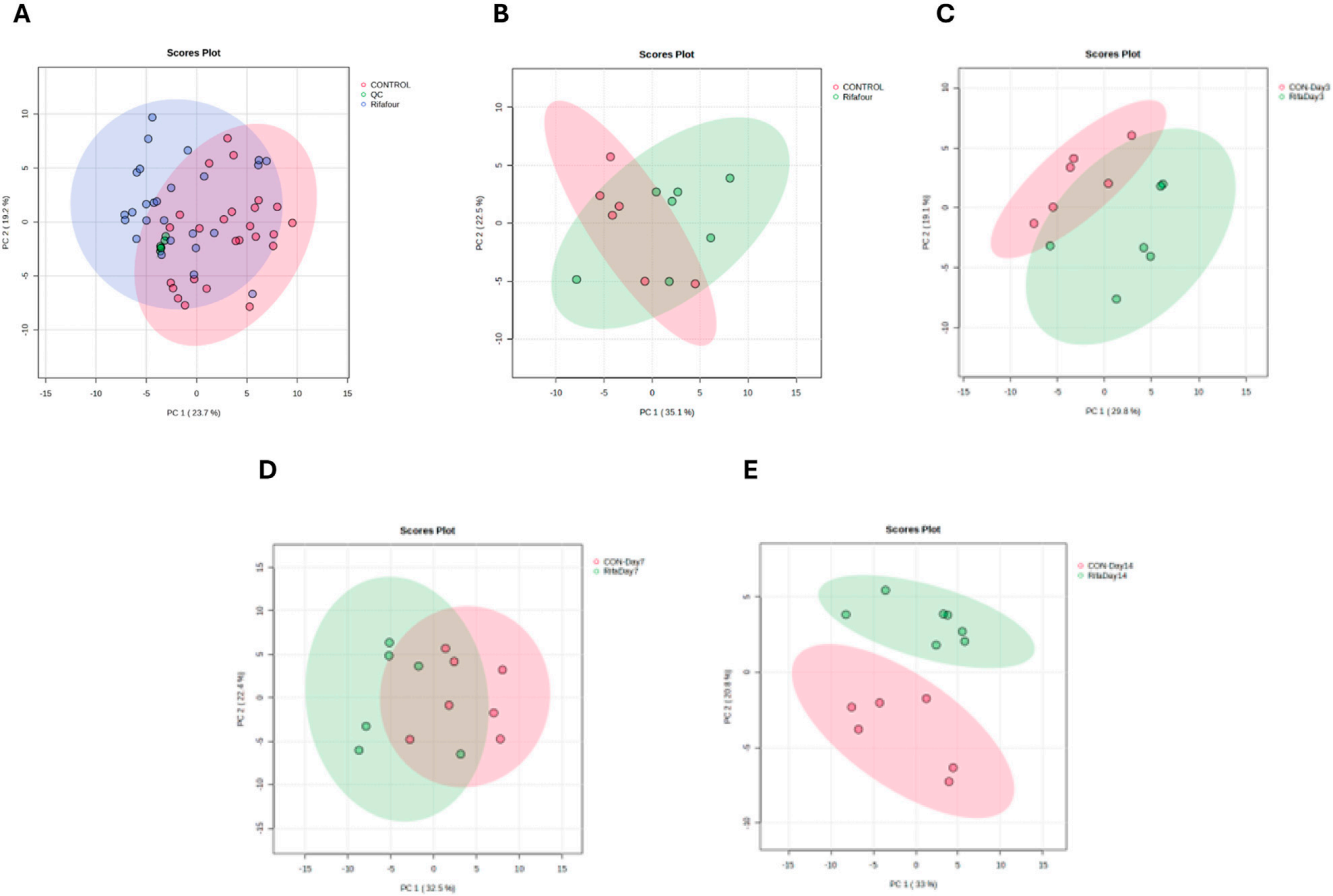
PCA score plots of urine samples. **(A)** All time points for the control and TB treatment groups, including quality control samples (QCs) that indicate reliable analytical data. The other PCA score plots show urine collected on day 1 **(B)**, day 3 **(C)**, day 7 **(D)**, and day 14 **(E)**. On day 1 (with no effect of TB treatment), there is a considerable overlap between the two groups. The trend here is that the differences between the two groups increase (greater separation) as the days progress to day 14.

Figure 2 shows the PCA score plots of all urine samples. Figure 2A provides an overview of the metabolic profiles of all urine samples from control, Rifafour-treated, and quality control (QC) groups. The clustering of the QCs demonstrates minimal analytical variability, ensuring the reliability and reproducibility of the data. The PCA and PLS-DA models demonstrate grouping across treated and control groups, indicating disparities, with statistical significance corroborated by variable importance in projection (VIP >1.0) and FDR-adjusted p-values <0.05 for the principal metabolites driving separation. No outliers were detected.

Figure 2B – day 1, illustrates the baseline metabolic profiles of all groups before Rifafour administration. The clustering on the PCA indicates a homogenous metabolic state, confirming the absence of treatment effects at this stage and providing a reference for subsequent comparisons. Figure 2C – day 3, shows a clear divergence between the control and Rifafour-treated groups, reflecting the early metabolic responses to the drug. These changes suggest initial systemic impacts of Rifafour on the host’s metabolism, potentially driven by acute biochemical adaptations. On day 7 (Figure 2D), the metabolic profiles of Rifafour-treated samples exhibit further divergence from those of the controls, indicating sustained metabolic alterations as the treatment progresses. By day 14 (Figure 2E), the separation between the treated and control groups becomes more pronounced, suggesting that Rifafour has overlapping effects on the metabolic pathways. These observations underscore the drugs’ ongoing impact on systemic metabolic dynamics.

**FIGURE 3:**
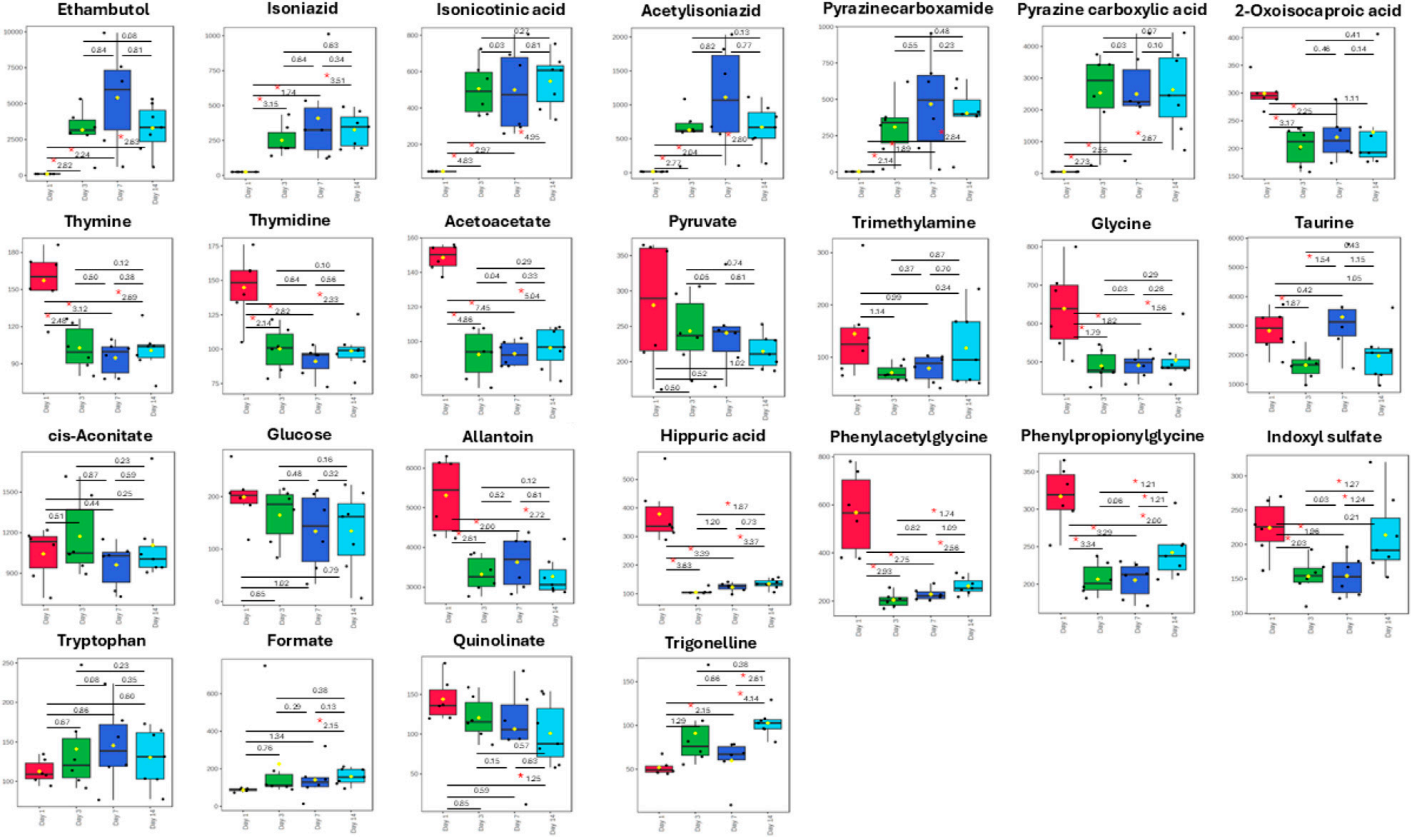
Box plots of important metabolites (VIP >1.0) identified in urine samples over 14 days of TB treatment. Anti-TB medication metabolites (ethambutol, isoniazid, isonicotinic acid, acetylisoniazid, pyrazinecarboxamide, pyrazine carboxylic acid) remained significantly elevated in the urine, as expected, during the 14 days of treatment. Statistical significance (FDR p-value <0.05) is indicated by a red star * and Hedge’s g-value given per group time comparison (g > 0.8 is a large effect size). Time points (day 1, 3, 7, or 14) are given on the x-axis. Concentrations (y-axis) given as mmol per mol creatinine.

Several metabolites were significantly altered between the Rifafour and control groups over the 14-day treatment (Figure 3). The box plots show examples of host metabolites and drug-derived metabolites that remained consistently perturbed over time. No conspicuous difference was observed in the urine samples obtained from both the control and the experimental groups on day 1. The similarity in metabolite concentration in the samples thus confirms that subsequent changes can be attributed to Rifafour administration. The concentration of allantoin ranged between 4,500 and 6,200 mmol per mol creatinine, and taurine ranged between 2,200 and 3,500 mmol per mol creatinine on day 1 from the treated and control samples. On day 1, phenylacetylglycine concentration was below 1,000 mmol per mol creatinine in samples obtained from both groups. Similarity was observed in the concentration range of most of the other identified metabolites on day 1, including trigonelline, quinolinate, formate, tryptophan, thymine, glucose, and hippuric acid.

When compared to day 1, decreases were observed in the concentration of metabolites in the treated groups on day 3, such as allantoin and taurine, which changed to the range of about 2,800 to 3,200 mmol per mol creatinine and 1,000 to 1,800 mmol per mol creatinine, respectively. However, an increase was seen in cis-aconitate concentration. Slight changes were observed in amino acids (such as glycine), depicting the activation of stress-response and detoxification pathways; these responses signify acute impacts of Rifafour metabolism.

On day 7, a further divergence from the control samples was seen, revealing moderate effects of Rifafour. Differences were observed in metabolites related to amino acid catabolism and lipid metabolism (ketone bodies), indicating adaptation to Rifafour administration/exposure. When compared, the treated samples from day 7 showed an increased concentration of the drug metabolites: ethambutol, Isoniazid, acetyl isoniazid, and pyrazine carboxylic acid. A decrease was observed in the concentrations of cis-aconitate, pyruvate, glycine, and phenylacetylglycine on day 7 compared to the concentrations in the treated samples on day 3 and day 1. Ongoing systemic adaptation to Rifafour administration was shown at this stage.

Day 14 revealed a pronounced difference in the metabolic profiles of the Rifafour-treated mice compared to the control group. A greater increase in the drug metabolites was observed in the mice, as expected, with alterations in the tricarboxylic acid cycle (TCA) intermediates, cis-aconitate and pyruvate. The sustenance of metabolites like ethambutol, isoniazid, 2-oxoisocaproic acid, pyrazine carboxylic acid, pyrazine carboxamide, and other drug metabolites reflects overlapping changes.

Within the control group, relative stability was observed in the metabolites across the time points, confirming a consistent baseline metabolic state. In contrast, significant time-dependent changes were observed in the treated group, starting with an early rise in the concentration of detoxification markers, such as glycine, and progressing to sustained changes in lipid and energy metabolism pathways, as indicated by the levels of pyruvate and glucose.

### 3.3 ^1^H-NMR results of feces

**FIGURE 4:**
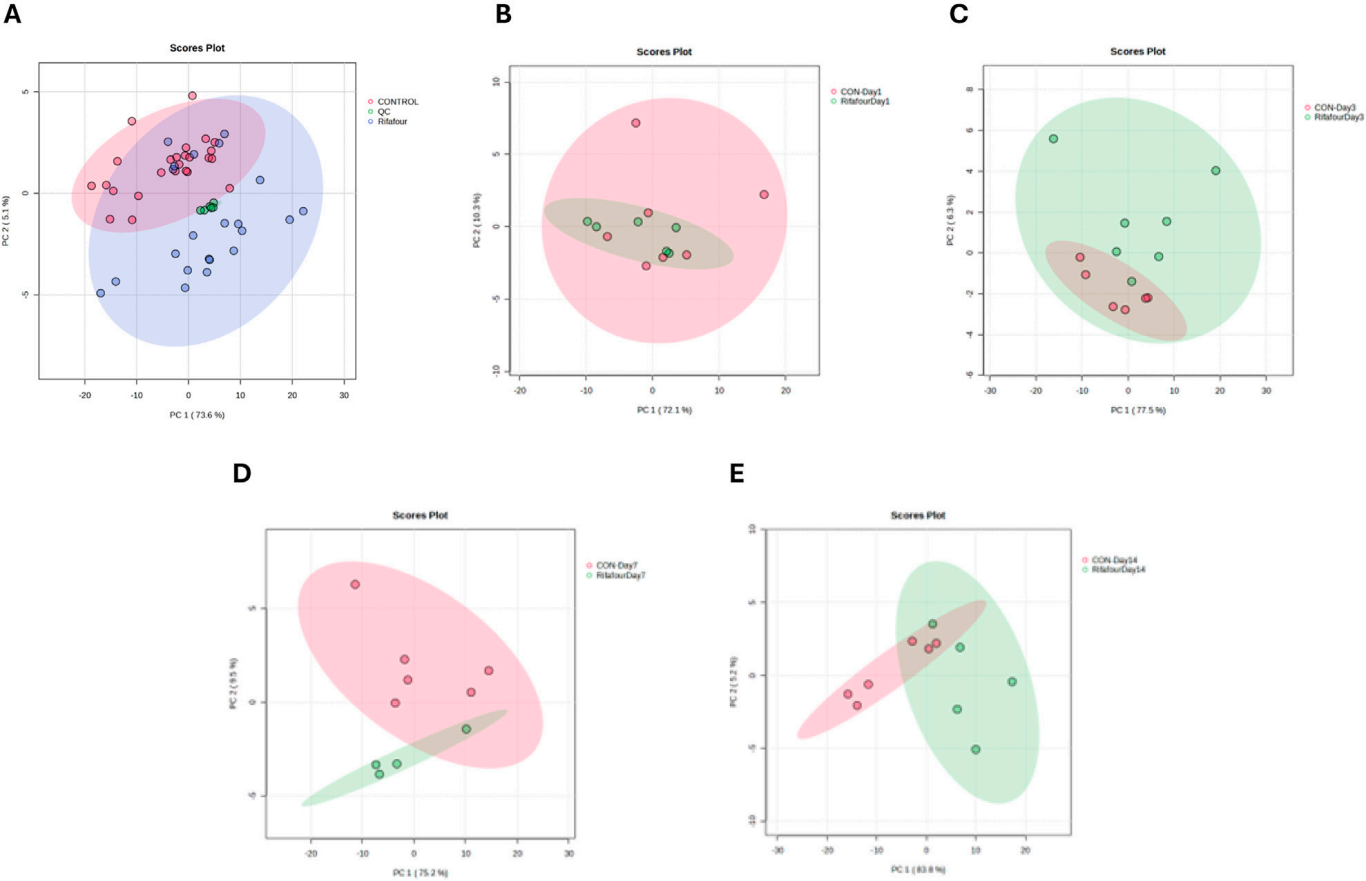
PCA score plots of fecal samples. **(A)** All time points for control and TB treatment groups, including quality control samples (QCs) that indicate reliable analytical data. The other PCA score plots show fecal samples on days 1 **(B)**, 3 **(C)**, 7 **(D)**, and 14 **(E)**. On day 1 (with no effect of TB treatment), there is a considerable overlap between the two groups. The trend here is that the differences between the two groups increase (greater separation) as the days progress to day 14.

Figure 4A shows a tight clustering of the QC samples, indicating negligible analytical drift and affirming the consistency and reliability of the analytical data. No clear outliers were detectable. The data obtained from day 1 (Figure 4B) of the experiment shows clustering of the control and treated samples together, as these are baseline samples. Figure 4C shows a slight divergence between the treated and control samples, thus suggesting the initial metabolic effect of Rifafour. Separation along PC1 and PC2 also suggests metabolic changes associated with the early metabolism of Rifafour. Overlap in some areas, as seen in the PCA plots, reflects some similarities in metabolic profiles between the control and treated groups on the third day (Figure 4C). A clearer distinction between the treated samples and the control is observed on day 7 (Figure 4D), indicating sustained and more notable metabolic alterations that can be associated with Rifafour. The samples treated form a distinct cluster, which stipulates systemic and consistent metabolic changes. On day 14 (Figure 4E), at the end of the experiment, continuous separation was observed, indicating an increasing metabolic disturbance caused by the drugs.

**FIGURE 5:**
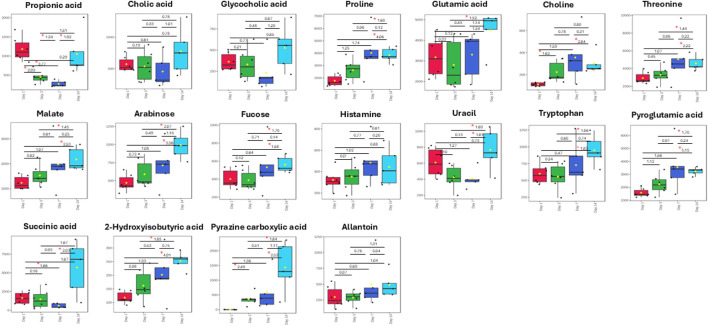
Box plots of important metabolites (VIP > 1.0) identified in fecal samples over 14 days of TB treatment. The only anti-TB medication metabolite that was detected in the feces was pyrazine carboxylic acid, which increased in concentration in the feces over the 14 days of treatment. Statistical significance (FDR p-value <0.05) is indicated by a red star * and Hedge’s g-value given per group time comparison (g > 0.8 is a large effect size). Time points (day 1, 3, 7, or 14) are given on the x-axis. Concentrations (y-axis) given as micromole per L or µM.

The box plots (Figure 5) show the changes in concentration of major metabolites (VIP >1.0) in the fecal samples of both the control and Rifafour-treated groups. These differences represent the biochemical transformation induced by the administration of Rifafour. The baseline samples (day 1) show similar metabolite levels between the control and treated groups. For all the VIP metabolites (VIP >1.0), overlapping median and interquartile ranges with slight variability were seen across the samples. This confirms the biochemical/metabolic homogeneity of the mice/groups before the administration of Rifafour.

By day 3, noticeable differences were observed in the treated samples compared to the controls; the metabolite concentrations varied, and while some of the metabolites had their concentrations elevated, others were decreased. Although not statistically significant, proline concentration increased from the range of 1,500 μM and 2,200 µM in the control samples to about 1,800 μM and 3,000 µM in the treated samples. Glutamic acid significantly increased from 1,900 μM and 3,800 µM in control samples to 1,900 μM and 4,500 µM in the treated samples. Choline concentration increased over treatment. A statistically significant surge was also observed in the concentrations of succinic acid, pyroglutamic acid, and glycocholic acid, while the concentrations of propionic acid and uracil decreased in the treated samples as compared to day 1. Significant metabolites in the treated samples include choline, succinic acid, glycocholic acid, proline, and fucose, which are host metabolites. Pyrazine carboxylic acid was the only observed drug metabolite in the feces. These metabolic changes indicate the onset of Rifafour metabolism. The surge in the concentration of pyrazine carboxylic acid may indicate increased production of drug-derived intermediates, while the decrease in the concentration of propionic acid suggests alteration of host-gut interactions (Zhang et al., 2023).

On Day 7, reductions in glutamic acid and succinic acid, along with an increase in choline and threonine, were observed in the feces (Figure 5). By Day 14, an increase in bile acid metabolites, propionic acid, succinic acid, and pyrazine carboxylic acid was seen, while threonine, glutamic acid, pyroglutamic acid, and proline decreased. A slight increase was observed in the concentration of arabinose, fucose, and histamine. 2-Hydroxyisobutyric acid returned to baseline levels.

Across all time points, minimal variability was seen in the control samples, underscoring the consistency of the metabolic profiles of the Kramnik mice in the absence of Rifafour treatment, thus supporting the results that the changes observed in the treatment samples can be attributed to Rifafour.

## 4 Discussion

This study presents a novel perspective on the systemic metabolic disruptions induced by the fixed-dose anti-TB drug Rifafour in a non-infected C3HeB/FeJ (Kramnik) mouse model. Despite the frequent use of Rifafour in clinical practice for the management of TB, its effects on host metabolism in the absence of infection have remained underexplored. By profiling urine, serum, and fecal samples across different time points (days 1, 3, 7, and 14), this study characterizes the dynamic metabolic shifts induced by Rifafour.

The multivariate PCA analysis performed on the three biofluids exhibited distinct separation between the treatment and control groups, especially from day 3 onwards. This clustering illustrates statistically significant metabolic disparities across the groups, corroborated by VIP >1.0 and FDR-adjusted p < 0.05 values, hence indicating the systemic and temporal influence of Rifafour on host metabolism. The clustering of quality control (QC) samples supports the reproducibility and robustness of the NMR dataset and analytical procedures. The metabolites of Rifafour (ethambutol, isoniazid, isonicotinic acid, acetylisoniazid, pyrazinecarboxamide, pyrazine carboxylic acid) were predominantly found in the urine and were found to be consistently elevated, indicating that, although these drugs are hepatotoxic, the mice maintained liver biotransformation of Rifafour over the 14 days. Pyrazine carboxylic acid was the only Rifafour metabolite present in the feces. Hence, consideration of the continued metabolism of Rifafour over the 14-day period is important to include in the multivariate model.

Serum metabolomics revealed significant differences between treated and control mice by day 14. The presence of glucose, taurine, glycine, uracil, histamine, and allantoin as key altered metabolites indicates disturbances across several metabolic domains. Also, the decrease in taurine and glycine may indicate altered antioxidant and detoxification pathways. The elevation of circulating glucose may result from hepatocellular dysfunction and impaired glycolytic regulation, as drug-induced mitochondrial stress can reduce hepatic glucose utilization (Tostmann et al., 2008; Ramappa and Aithal, 2013; Singh et al., 2015). Similarly, altered amino acid levels (such as glycine and tryptophan) may reflect increased protein catabolism and impaired urea cycle function, both of which are characteristic of liver injury induced by isoniazid and rifampicin (Ramappa and Aithal, 2013; Nhung et al., 2025). Collectively, these changes are consistent with hepatotoxic stress mechanisms previously described for first-line anti-TB drugs.

The early reduction in allantoin and taurine in the urine on day 3, both of which are antioxidants and metabolites associated with detoxification, may indicate an acute oxidative stress response to Rifafour metabolism (Tostmann et al., 2008; Ramappa and Aithal, 2013). The elevation of drug-derived metabolites, particularly isoniazid and pyrazine carboxylic acid, substantiates active drug absorption and elimination at this first phase (Van Zyl et al., 2020). By Day 7, reductions in amino acids like glycine and phenylacetylglycine reflect modifications in nitrogen metabolism and host detoxification mechanisms (Ramappa and Aithal, 2013), while variations in cis-aconitate and pyruvate signify disruptions in the TCA cycle (Nhung et al., 2025). By Day 14, the sustained presence of modified TCA intermediates (pyruvate, cis-aconitate) and detoxifying metabolites in the urine signifies a transition to long-term metabolic adaptation due to ongoing Rifafour exposure, underscoring potential hepatotoxic stress and energy dysregulation (Singh et al., 2015).

The elevation of pyruvate and drug-specific metabolite concentrations (such as pyrazine carboxylic acid) indicates the rapid absorption and systemic circulation of Rifafour components. The sustained changes in pyruvate and cis-aconitate indicate disruptions in the TCA cycle and energy metabolism. Similar disturbances have been associated with mitochondrial stress due to isoniazid exposure (Nhung et al., 2025).

In the fecal metabolome, the sustained elevation of pyrazine carboxylic acid in feces confirms active drug metabolism and excretion, while changes in microbial metabolites such as succinic acid and propionic acid signify disrupted microbial metabolic networks, which is consistent with known broad-spectrum antibiotic effects (Ferreyra et al., 2014). On Day 7, reductions in glutamic acid and succinic acid, both associated with energy metabolism and microbial fermentation, may indicate a disturbance in gut microbial activity or compromised host energy mechanisms due to Rifafour pressure (Jiang and Schnabl, 2020; Qi et al., 2020). The increase in choline at this stage indicates an adaptive hepatic response, given that choline is crucial for phospholipid metabolism and liver protection (Jiang and Schnabl, 2020). The elevation of threonine, an important amino acid, may signify augmented protein degradation and stress-induced catabolism (Macelline et al., 2021). By Day 14, the increase in bile acid metabolites, including glycocholic acid and cholic acid, further highlights potential drug-induced disruptions in gut-liver axis interactions (Zhang et al., 2023).

Choline and pyrazine carboxylic acid showed a sustained elevation, with an increase in variation observed (within the interquartile range) in the treated samples during the later days of Rifafour administration, compared to the earlier days. Sustained choline elevation depicts that there was an adaptation/insignificant impairment to its pathways and the efficient regulation of the choline pathway (transport, utilization, and acetylcholine synthesis). The observed high level of threonine suggests a continuous breakdown of proteins and release of amino acids, which can signify impaired liver function or Rifafour-induced stress (Macelline et al., 2021). Depletion of glutamic acid and succinic acid may be associated with drug-induced stress, liver dysfunction, metabolic dysregulation, and altered gut microbial composition (Qi et al., 2020; Jiang and Schnabl, 2020). The increase in choline, particularly by day 14, suggests a potential disruption of phospholipid and hepatic pathways.

Time point analysis of metabolomic shifts reveals a distinct progression of host responses to Rifafour: an initial phase characterized by oxidative stress and detoxification requirements (Day 3), a mid-treatment phase marked by modifications in amino acid, nitrogen, and energy metabolism (Day 7), and a final phase defined by overlapping disruptions in bile acid metabolism and the gut–liver axis (Day 14). This temporal pattern underscores the immediate and gradual systemic effects of Rifafour, highlighting the significance of treatment duration in assessing host metabolism and drug-related toxicity. These molecular insights indicate that monitoring glucose and amino acid levels during Rifafour therapy may yield early signs of hepatotoxic stress, thereby providing prospective metabolic markers for patient safety and medication optimization in tuberculosis management.

Our results align with recent multi-omic and metabolomic research indicating that rifampicin, frequently used in conjunction with isoniazid, interferes with central metabolic pathways, including glucose, lipid, and amino acid metabolism, oxidative stress, and energy balance. An integrated proteomic and metabolomic analysis in mice administered isoniazid and rifampicin revealed extensive dysregulation of 31 metabolites and 511 proteins, indicating disrupted lipid and amino acid metabolism, as well as oxidative stress, inflammation, and early steatosis (Song et al., 2022). A further metabolomics investigation highlighted modifications in fatty acid metabolism and the TCA cycle due to INH/RIF-induced hepatotoxicity in murine models (Ke et al., 2018). Additionally, mechanistic investigations in murine hepatic tissue indicated that rifampicin induces the upregulation of genes associated with fatty acid synthesis (e.g., Fas, Acc, Scd-1) and activates omega-oxidation pathways (Cyp4a10, Cyp4a14), thereby supporting our findings of lipid and bile acid disturbances (Huang et al., 2016).

While numerous previous studies concentrated on serum or hepatic metabolism, our research broadens this scope by revealing alterations in the fecal metabolome, specifically in bile acids and gut–liver axis-related markers, highlighting a more holistic systemic effect of rifampicin-containing therapy that encompasses gut microbial and biliary metabolism. In contrast to previous studies, which have mostly examined serum or hepatic metabolism, our research provides more insights into modifications in the fecal metabolome, specifically regarding bile acids and microbial-associated metabolites. This underscores the significance of the gut–liver axis in the systemic metabolic effects generated by Rifafour, enhancing the existing comprehension of rifampicin metabolism.

## 5 Conclusion

This study shows the systemic metabolic influences of Rifafour (an anti-TB medication) in the urine, feces, and serum of healthy Kramnik (C3HeB/FeJ) mice. The purpose of this study was to identify the metabolites perturbed by Rifafour, so that when analyzing Rifafour-treated mice with *M. tb* infection, the metabolites identified here can be subtracted from the analyses. Hence, removing the metabolite affected by the treatment itself allows future studies to focus on host metabolism in response to *M. tb*. Several limitations are worth noting in this study. As in all animal studies, the translatability to humans can vary. Larger sample sizes are needed to support the findings of this study. Some mechanistic claims (e.g., taurine/glycine depletion indicating oxidative stress/hepatotoxicity; pyruvate/cis-aconitate shifts because of mitochondrial suppression) are plausible but not directly verified in this study. Hence, it is worth noting that our discussion is primarily hypothesis-generating. While ^1^H-NMR spectroscopy is reproducible and robust, it is limited in sensitivity compared to mass spectrometry (MS)-based platforms. Hence, combining ^1^H-NMR and MS metabolomics approaches would increase the number of identified and quantified metabolites, especially for biomarkers with low abundance. Moreover, diversity in metabolic responses may stem from strain-specific genetic variables, exemplified by the distinct susceptibility profile of C3HeB/FeJ mice, as well as from variations in medication dosage or treatment duration. Subsequent research may investigate whether the metabolites identified herein (e.g., taurine, glycine, and choline) can serve as early indicators of drug-induced hepatotoxicity and further analyze the gut–liver axis by integrating metabolomics with microbiome analysis. Comparative analyses among various mouse strains, genetic backgrounds, or differing treatment protocols may yield a deeper understanding of host–drug interactions and augment the translational significance of these results. Therapeutically, the monitoring of metabolites such as taurine, glycine, and choline may provide early metabolic indicators of hepatotoxic stress during Rifafour treatment. Moreover, supplementary measures designed to maintain antioxidant defenses and bile acid equilibrium, such as antioxidant supplements or hepatoprotective adjuvants, should be contemplated in forthcoming preclinical and clinical studies to alleviate treatment-related toxicity.

## Data Availability

The raw data supporting the conclusions of this article will be made available by the authors, without undue reservation.

## References

[B1] Blomberg B. Spinaci S. Fourie B. Laing R. (2001). The rationale for recommending fixed-dose combination tablets for treatment of tuberculosis. Bull. World Health Organ. 79, 61–68. 11217670 PMC2566330

[B2] Catussi B. L. C. Turco E. G. L. Pereira D. M. Teixeira R. M. N. Castro B. P. Massaia I. F. D. (2024). Metabolomics: unveiling biological matrices in precision nutrition and health. Clin. Nutr. ESPEN 64, 314–323. 10.1016/j.clnesp.2024.10.148 39427750

[B3] Davoren E. Mason S. (2023). ^1^H-NMR protocol for rapid diagnosis of purine and pyrimidine metabolic disorders in urine. Star. Protoc. 4, 102181. 10.1016/j.xpro.2023.102181 36961819 PMC10060748

[B4] Deshpande A. Likhar R. Khan T. Omri A. (2024). Decoding drug resistance in mycobacterium tuberculosis complex: genetic insights and future challenges. Expert Rev. Anti-infective Ther. 22, 511–527. 10.1080/14787210.2024.2400536 39219506

[B5] Driver E. R. Ryan G. J. Hoff D. R. Irwin S. M. Basaraba R. J. Kramnik I. (2012). Evaluation of a mouse model of necrotic granuloma formation using C3HeB/FeJ mice for testing of drugs against *Mycobacterium tuberculosis* . Antimicrob. Agents Chemother. 56 (6), 3181–3195. 10.1128/AAC.00217-12 22470120 PMC3370740

[B6] Farhat M. Cox H. Ghanem M. Denkinger C. M. Rodrigues C. Abd El Aziz M. S. (2024). Drug-resistant tuberculosis: a persistent global health concern. Nat. Rev. Microbiol. 22 (10), 617–635. 10.1038/s41579-024-01025-1 38519618

[B7] Ferreyra J. A. Wu K. J. Hryckowian A. J. Bouley D. M. Weimer B. C. Sonnenburg J. L. (2014). Gut microbiota-produced succinate promotes C. difficile infection after antibiotic treatment or motility disturbance. Cell Host Microbe 16, 770–777. 10.1016/j.chom.2014.11.003 25498344 PMC4859344

[B8] Huang J.-H. Zhang C. Zhang D.-G. Li L. Chen X. Xu D.-X. (2016). Rifampicin-induced hepatic lipid accumulation: association with up-regulation of peroxisome proliferator-activated receptor γ in mouse liver. PloS One 11, e0165787. 10.1371/journal.pone.0165787 27806127 PMC5091861

[B9] Jiang L. Schnabl B. (2020). Gut microbiota in liver disease: what do we know and what do we not know? Physiology 35, 261–274. 10.1152/physiol.00005.2020 32490750 PMC7474259

[B10] Ke X.-H. Wang C.-G. Luo W.-Z. Wang J. Li B. Lv J.-P. (2018). Metabolomic study to determine the mechanism underlying the effects of Sagittaria sagittifolia polysaccharide on isoniazid-and rifampicin-induced hepatotoxicity in mice. Molecules 23, 3087. 10.3390/molecules23123087 30486347 PMC6321494

[B11] Li Y. Yao C. C. Ji P. Wang H. Y. Wang S. J. Wang Y. (2025). Plasma dynamic metabolomics during anti-tuberculosis treatment identifies an amino acid panel with diagnostic potential. LabMed Discov. 2 (1), 100059. 10.1016/j.lmd.2025.100059

[B12] Lin C. Tian Q. Guo S. Xie D. Cai Y. Wang Z. (2024). Metabolomics for clinical biomarker discovery and therapeutic target identification. Molecules 29, 2198. 10.3390/molecules29102198 38792060 PMC11124072

[B13] Macelline S. P. Chrystal P. V. Liu S. Y. Selle P. H. (2021). Implications of elevated threonine plasma concentrations in the development of reduced-crude protein diets for broiler chickens. Animal Prod. Sci. 61, 1442–1448. 10.1071/an20554

[B14] Mason S. Terburgh K. Louw R. (2018). Miniaturized 1H-NMR method for analyzing limited-quantity samples applied to a mouse model of Leigh disease. Metabolomics 14, 74–12. 10.1007/s11306-018-1372-6 30830372

[B15] Nhung T. T. M. Phat N. K. Anh T. T. Nhgi T. D. Thu N. Q. Lee A. (2025). Endoplasmic reticulum stress inhibition preserves mitochondrial function and cell survival during the early onset of isoniazid-induced oxidative stress. Chemico-Biological Interact. 411, 111448. 10.1016/j.cbi.2025.111448 40015660

[B16] Qi X. Yang M. Stenberg J. Dey R. Fogwe L. Alam M. S. (2020). Gut microbiota mediated molecular events and therapy in liver diseases. World J. Gastroenterology 26, 7603–7618. 10.3748/wjg.v26.i48.7603 33505139 PMC7789060

[B17] Ramappa V. Aithal G. P. (2013). Hepatotoxicity related to anti-tuberculosis drugs: mechanisms and management. J. Clin. Exp. Hepatology 3, 37–49. 10.1016/j.jceh.2012.12.001 25755470 PMC3940184

[B19] Singh A. Prasad R. Balasubramanian V. Gupta N. Gupta P. (2015). Prevalence of adverse drug reaction with first-line drugs among patients treated for pulmonary tuberculosis. Clin. Epidemiol. Glob. Health 3, S80–S90. 10.1016/j.cegh.2015.10.005

[B20] Song Y. Qu X. Tao L. Gao H. Zhang Y. Zhai J. (2022). Exploration of the underlying mechanisms of isoniazid/rifampicin‐induced liver injury in mice using an integrated proteomics and metabolomics approach. J. Biochem. Mol. Toxicol. 36, e23217. 10.1002/jbt.23217 36111668

[B21] Thirion A. Loots D. T. Williams M. E. Solomons R. Mason S. (2024). 1H-NMR metabolomics investigation of CSF from children with HIV reveals altered neuroenergetics due to persistent immune activation. Front. Neurosci. 18, 1270041. 10.3389/fnins.2024.1270041 38745940 PMC11091326

[B22] Tong L. Feng Q. Lu Q. Zhang J. Xiong Z. (2022). Combined 1H NMR fecal metabolomics and 16S rRNA gene sequencing to reveal the protective effects of Gushudan on kidney-yang-deficiency-syndrome rats via gut-kidney axis. J. Pharm. Biomed. Analysis 217, 114843. 10.1016/j.jpba.2022.114843 35623116

[B23] Tostmann A. Boeree M. J. Aarnoutse R. E. De Lange W. C. Van Der Ven A. J. Dekhuijzen R. (2008). Antituberculosis drug‐induced hepatotoxicity: concise up‐to‐date review. J. Gastroenterology Hepatology 23, 192–202. 10.1111/j.1440-1746.2007.05207.x 17995946

[B24] Van Zyl C. D. W. Loots D. T. Solomons R. Van Reenen M. Mason S. (2020). Metabolic characterization of tuberculous meningitis in a South African paediatric population using 1H NMR metabolomics. J. Infect. 81, 743–752. 10.1016/j.jinf.2020.06.078 32712206

[B25] Zhang Q. Hu W.-M. Deng Y.-L. Wan J.-J. Wang Y.-J. Jin P. (2023). Dysbiosis of gut microbiota and decreased propionic acid associated with metabolic abnormality in Cushing’s syndrome. Front. Endocrinol. 13, 1095438. 10.3389/fendo.2022.1095438 36755580 PMC9901362

